# Supplementary material for: Impaired gastric and urinary but preserved cardiac interoception in women with endometriosis
Source: PLoS One. 2025 May 5;20(5):e0322865. doi: 10.1371/journal.pone.0322865 (PMC12052146; doi:10.1371/journal.pone.0322865)
Supplement: S1 File — File including all supplementary figures, tables, and analyses. (PDF) [file pone.0322865.s001.pdf]

Impaired gastric and urinary but preserved cardiac interoception in women with endometriosis

Chiara Cantoni[1*], Sofia Ciccarone[1], Maria Grazia Porpora[2] and Salvatore Maria Aglioti[1,3,4]

[1] Department of Psychology, Sapienza University of Rome, Rome, Italy.

[2] Department of Maternal, Infantile and Urological Sciences, Sapienza University of Rome, Rome, Italy.

[3] Department of Psychology, Sapienza University of Rome & Center for Life Nano- & Neuroscience, Italian Institute of Technology (IIT), Rome, Italy.

[4] IRCCS Santa Lucia Foundation, Rome, Italy.

*Corresponding author:
E-mail: chiara.cantoni@uniroma1.it (CC)

Supplementary materials

Supplementary Figures S1, S2

S1 Fig. Heartbeat Counting Task.

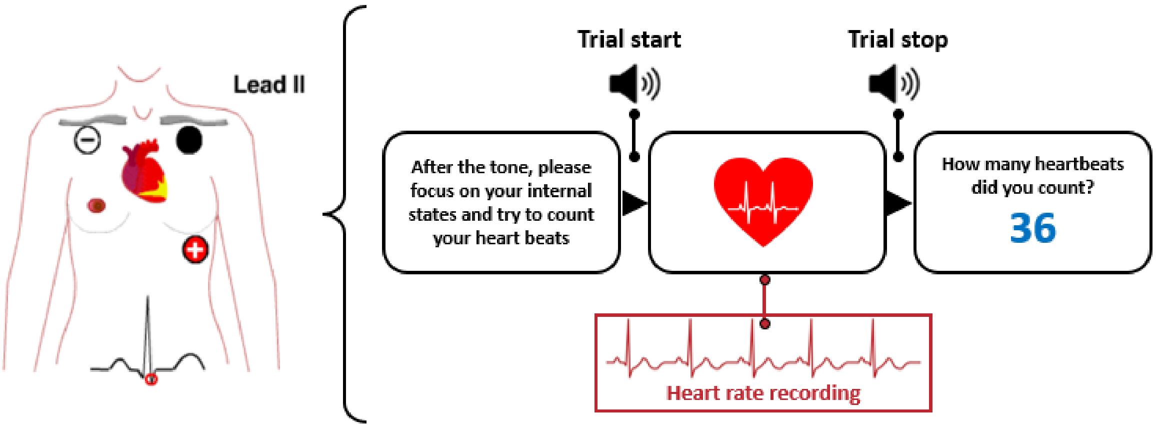

Graphical representation of the Heartbeat Counting Task by Schandry (1981).

S2 Fig. Two-step Water Load Test.

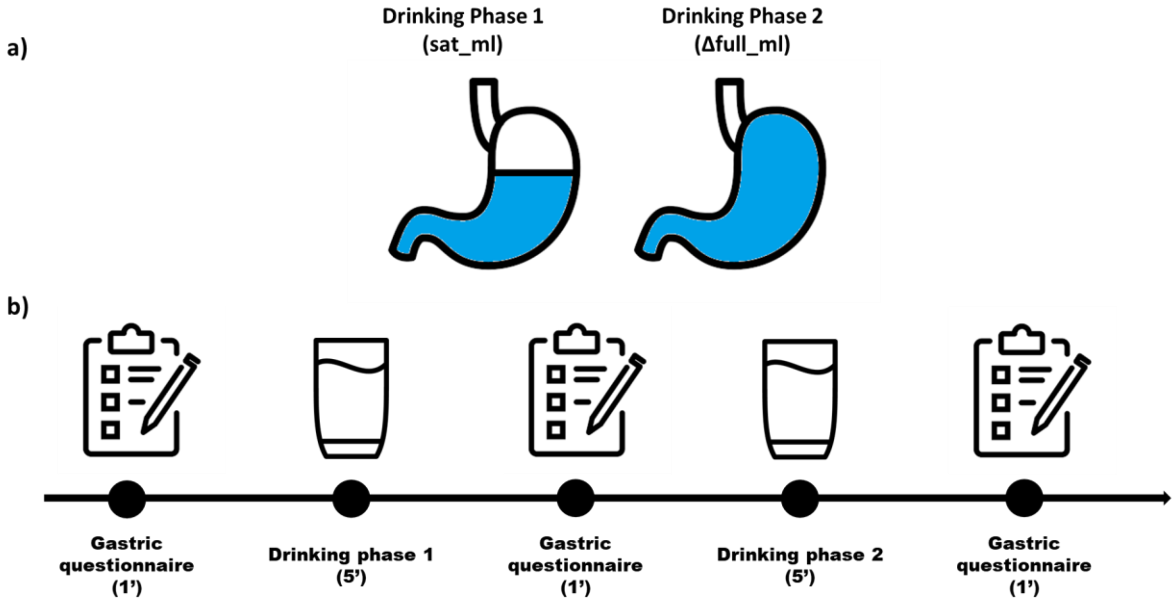

Graphical description of the gastric Two-step Water Load Test by van Dyck and colleagues (2016). a) Drinking phase 1 (satiety) and drinking phase 2 (fullness); b) Task procedure.

Interoceptive Accuracy Tasks

The following questionnaire, adapted from van Dyck et al. (2016), assesses gastric sensations. The questionnaire was completed at the beginning of the task (e.g., baseline), after the first drinking phase (e.g., satiety) and after the second drinking phase (e.g., fullness).

S1 Table. Gastric water load questionnaire.

Q1: How satiated do you feel right now?	1	2	3	4	5	6	7
Q2: How full do you feel right now?	1	2	3	4	5	6	7
Q3: How much discomfort do you feel right now?	1	2	3	4	5	6	7
Q4: How much guilt do you feel right now?	1	2	3	4	5	6	7
Q5: How sluggish do you feel right now?	1	2	3	4	5	6	7
Q6: How much nausea do you have right now?	1	2	3	4	5	6	7
Q7: How aroused do you feel right now?	1	2	3	4	5	6	7

Gastric water load questionnaire assessing gastric sensations.

S2 Table. Descriptive statistics of the Gastric Water Load.

	CONTROL SUBJECTS			PATIENTS WITH ENDOMETRIOSIS		
	**BASELINE**	**SATIETY**	**FULLNESS**	**BASELINE**	**SATIETY**	**FULLNESS**
**Q1**	3.20±1.86	5.60±1.48	6.23±1.17	3.73±1.82	5.77±1.36	6.23±1.07
**Q2**	***2.37±1.54***	5.37±1.47	6.63±0.67	***3.50±1.74***	5.63±1.40	6.67±0.61
**Q3**	2.23±1.57	***2.53±1.53***	3.73±1.84	2.83±2.10	***3.60±1.94***	4.37±2.04
**Q4**	***1.27±0.74***	***1.17±0.59***	***1.20±0.71***	***2.57±2.13***	***2.13±1.70***	***1.96±1.50***
**Q5**	2.87±1.80	2.40±1.52	2.50±1.61	3.83±2.00	3.17±1.64	3.23±1.57
**Q6**	***1.03±0.41***	1.77±1.25	2.47±1.87	***1.77±1.33***	2.10±1.54	3.27±1.91
**Q7**	4.03±1.40	4.40±1.52	4.27±1.84	3.73±1.51	4.03±1.30	3.57±1.28

Means and Standard Deviations of the Gastric water load questionnaire assessing gastric sensations across the experiment (i.e., at baseline, after the first session to reach satiety and after the second session to reach fullness) for healthy controls and women with endometriosis. Differences in the means between the two groups are marked in bold.

Novel Urinary Interoceptive Task - Questionnaire assessing participant's attention and concern for their bladder stimuli:

S3 Table. Urinary water load questionnaire.

In your daily life, how much do you pay attention to the stimuli coming from your bladder?	1	2	3	4	5	6	7	8	9	10
In your daily life, how much do the stimuli from your bladder interfere with the activities you have to perform?	1	2	3	4	5	6	7	8	9	10
In your daily life, how much do you worry about the stimuli coming from your bladder?	1	2	3	4	5	6	7	8	9	10
In your daily life, how often do you wake up during the night due to stimuli from your bladder?	1	2	3	4	5	6	7	8	9	10
In your daily life, how often during the day do you feel the need to go to the bathroom to empty your bladder?	1	2	3	4	5	6	7	8	9	10

Urinary water load questionnaire assessing urinary sensations.

To perform both gastric and urinary water load we used natural water with the following characteristics: Source water temperature: 6.0 °C; Specific electrical conductivity at 20 °C: 69.0 µS/cm; Total hardness in French degrees: 3.9; pH at source: 7.1; Fixed residue at 180°: 50.2 mg/l; Free carbon dioxide at source: 5.0 mg/l; Calcium: 11.5 mg/l; Magnesium: 2.8 mg/l; Sulphates: 4.9 mg/l; Sodium: 1.2 mg/l; Potassium: 0.51 mg/l; Chlorides: 0.27 mg/l; Nitrites: less than 0.002 mg/l; Bicarbonates: 42 mg/l. Water bottles were stored in the same place to maintain, as far as possible, a fixed temperature at the time of drinking.

Description of the questionnaires

To measure interoceptive sensibility (i.e. subjective assessment of one's interoceptive abilities), participants were asked to complete:

Multidimensional Assessment of Interoceptive Awareness II (MAIA-II; Mehling et al., 2018): is a questionnaire consisting of eight subscales: Noticing (i.e., awareness of uncomfortable, comfortable, and neutral body sensations), Not-Distracting (i.e., tendency not to ignore or distract oneself from sensations of pain or discomfort), Not-Worrying (i.e., tendency not to worry or experience emotional distress with sensations of pain or discomfort), Attention Regulation (i.e., ability to sustain and control attention to body sensations), Emotional Awareness (i.e., awareness of the connection between body sensations and emotional states), Self-Regulation (i.e., ability to regulate distress by paying attention to body sensations), Body Listening (i.e., active listening to the body for insight), and Trusting (i.e., experience of one’s body as safe and trustworthy);

To assess participants’ perception of their body image and their body dissatisfaction the Body Uneasiness Test was administered:

Body Uneasiness Test (BUT; Cuzzolaro et al., 2006): is a questionnaire used to assess participants’ perception of their body image and their body dissatisfaction, consisting of two parts: “BUT_A” composed by five subscales (i.e., Weight Phobia, namely the fear of gain weight; Body Image Concerns, namely worries related to physical appearance; Avoidance, namely body image related avoidance behaviour; Compulsive Self-Monitoring, namely compulsive checking of physical appearance; and Depersonalization, namely detachment and estrangement feelings toward the body), and “BUT_B” which explores specific worries related to particular body parts or functions (e.g., head, thighs, legs, skin).

Further results

Relationship between the Interoceptive Tasks

Besides measuring correlations in the full sample (women with endometriosis and healthy controls), we also measured correlations between the novel urinary task and the cardiac and gastric tasks in the sample of healthy controls and patients with endometriosis separately. Results showed that the novel urinary water load did not correlate with the Heartbeat Counting Task (R=0.2389894, p=0.2034) but did correlate with the Water Load Test (R=0.5522, p= 0.001557) in the healthy control sample. Conversely, in the group of people with endometriosis, the novel urinary interoceptive task neither correlated with the Heartbeat Counting Task (R=0.1516019, p=0.4239) nor with the Water Load Test (R=0.1357286, p= 0.4745).

Interoceptive Sensibility Questionnaires

<u>*Multidimensional Assessment of Interoceptive Awareness II*</u> - Results of the t-test for each subscale of the MAIA-II questionnaire to assess differences in interoceptive sensibility between patients with endometriosis and healthy controls. Please note that for the *"Noticing"*, *"Trusting"* and *"Emotional Awareness"* subscales we performed Mann-Whitney non-parametric tests as data were not normally distributed: MAIA Noticing: U= 395, p = 0.413; MAIA Trusting: U= 371, p = 0.243; MAIA Body Listening: t(58) = -0.22038, p = 0.8263; MAIA Self-Regulation: t(58) = 0.36285, p = 0.718; MAIA Emotional Awareness U= 411, p = 0.567; MAIA Attention Regulation: t(58) = -0.14633, p = 0.8842; MAIA Not Worrying: t(58) = 1.7819, p = 0.08; MAIA Not Distracting: t(58) = 1.2403, p = 0.2199.

Body Image questionnaire

<u>*Body Uneasiness Test*</u> - Results of the t-tests for each subscale of the Body Uneasiness Test assessing differences in body image dissatisfaction between patients with endometriosis and healthy controls: BUT_Weight Phobia: t(57.999) = 0.98972, p = 0.3264; BUT_Body Image Concern: t(56.35) = -0.28482, p = 0.7768; BUT_Avoidance: t(50.538) = -0.30042, p = 0.7651; BUT_ Compulsive Self-Monitoring: t(57.905) = -0.90575, p = 0.3688; BUT_Depersonalization: t(46.951) = -2.2049, p =

0.0324; BUT_Testa: t(52.107)= 0.89379, p = 0.3755; BUT_Cosce: t(57.514) = -0.68991, p = 0.493; BUT_Gambe: t(54.897) = 1.0545, p = 0.2963; BUT_Pelle: t(57.926) = 0.6085, p = 0.5452.

Further analyses

To make sure that patients' low gastric and bladder interoceptive accuracy were not due to differences related to different treatments of endometriosis, we checked for any differences between patients who had or had not undergone surgery; interestingly, no differences in gastric (t(28)= -0.24944, p = 0.8048) or bladder (t(28)= -0.66266, p = 0.513) interoceptive accuracy emerged in these two subgroups. We also controlled for any hormonal medication taken (e.g., progestin, oestrogen, or no medication), and found that no significant differences in gastric (F(2,27)=1.516; p = 0.238) or bladder (F(2,27)=1.398; p = 0.265) interoceptive accuracy emerged in these three subgroups. We also checked that any differences between patients with endometriosis and healthy controls in bladder interoceptive accuracy were not due to differences in urinary incontinence or urinary retention. Therefore, we measured whether there was a difference between patients and control subjects in the time required to reach the maximum stimulus (VAS=10). No significant differences (t(58)= 1.7138, p = 0.09191) emerged.
