# Supplementary material for: Impaired gastric and urinary but preserved cardiac interoception in women with endometriosis
Source: PLoS One. 2025 May 5;20(5):e0322865. doi: 10.1371/journal.pone.0322865 (PMC12052146; doi:10.1371/journal.pone.0322865)
Supplement: Attachment — Submitted filename: Reviewers comments.docx [file pone.0322865.s002.docx]

The study compared interoceptive accuracy and interoceptive beliefs in women with endometriosis and healthy controls across cardiac, gastric, and urinary domains. Women with endometriosis showed reduced accuracy in gastric and urinary interoception but not cardiac interoception. Despite this, they reported heightened bladder interoceptive beliefs, characterised by increased attention and concern about bladder signals. Group marginally moderated the effect of chronic pain on urinary interoception, suggesting that heightened pain sensitivity may impair interoceptive accuracy. The novel Urinary Interoceptive Task correlated with gastric but not cardiac interoceptive accuracy, highlighting shared neural pathways for gastric and bladder signals.

This study highlights dissociations between objective interoceptive accuracy and subjective interoceptive beliefs in women with endometriosis, suggesting that interoceptive impairments may result from pain hypersensitivity and hypervigilance. The findings provide valuable insights for developing non-invasive diagnostic tools and interoceptive training therapies for chronic pain management. This study addresses a relevant topic in interoception and employed a well-designed method. The results are informative and important in further elucidating the relationship between chronic pain and interoception. The manuscript offers an interesting read and would make a meaningful contribution to the interoception and pain literature following revision and clarification of the following details:

Introduction

The terminology around interoceptive dimensions would benefit from updating to match the more recent multidimensional framework by Suksasilp & Garfinkel (2022), *Towards a comprehensive assessment of interoception in a multi-dimensional framework*, and change terms such as interoceptive sensibility to interoceptive beliefs, and interoceptive awareness to interoceptive insight, accordingly.

Method

POWER analysis: More detail is required regarding the test used. Please clarify which test family, statistical test, type of power analysis, and other relevant input parameters (e.g. number of tails) were entered.

Water load test:

How long apart were the two drinking phases; i.e., did drinking phase 2 follow phase 1 immediately?

State the meaning of the index regarding interoceptive accuracy (higher index, higher accuracy?)

Urinary interoception task:

On page 7, lines 172–174, you mention the use of a VAS for participants to rate their bladder sensations. Could you clarify in the manuscript which specific rating on the scale (0–10) was defined as the 'minimum urinary stimulus'?

To enhance the reliability and validity of the findings, please clarify whether the same subjective rating of the 'minimum urinary stimulus' was consistently applied within participants across the void2ml and void3ml conditions. For example, if a participant indicated '4' as the minimum in the void2ml condition, was the same rating ('4') considered as the minimum in the void3ml condition?

If consistency was not maintained, I recommend noting this as a limitation in the manuscript, given the importance of this measure in calculating the *Urinary Interoceptive Index*. Specifically, discuss how variability in subjective thresholds and baseline urinary sensations might have influenced condition comparability (e.g., void2ml vs. void3ml within participants), introduced noise into the data, or affected the interpretation of findings.

Line 183: Replace "her" with "their" to ensure inclusive language.
