# Supplementary material for: Impaired gastric and urinary but preserved cardiac interoception in women with endometriosis
Source: PLoS One. 2025 May 5;20(5):e0322865. doi: 10.1371/journal.pone.0322865 (PMC12052146; doi:10.1371/journal.pone.0322865)
Supplement: Attachment — Submitted filename: Response to Reviewers.docx [file pone.0322865.s004.docx]

If applicable, we recommend that you deposit your laboratory protocols in [protocols.io](http://protocols.io/) to enhance the reproducibility of your results. Protocols.io assigns your protocol its own identifier (DOI) so that it can be cited independently in the future. For instructions see: [https://journals.plos.org/plosone/s/submission-guidelines#loc-laboratory-protocols](https://track.editorialmanager.com/CL0/https:%2F%2Fjournals.plos.org%2Fplosone%2Fs%2Fsubmission-guidelines%23loc-laboratory-protocols/1/010f0194405aff49-a661a09f-0148-44f4-9fae-d9440c0c2932-000000/zqQHtMl92XUwpCrOHlT2rxE9ZDUT5dNvvLpjacH_IYg=192). Additionally, PLOS ONE offers an option for publishing peer-reviewed Lab Protocol articles, which describe protocols hosted on [protocols.io](http://protocols.io/). Read more information on sharing protocols at [https://plos.org/protocols?utm_medium=editorial-email&utm_source=authorletters&utm_campaign=protocols](https://track.editorialmanager.com/CL0/https:%2F%2Fplos.org%2Fprotocols%3Futm_medium=editorial-email%26utm_source=authorletters%26utm_campaign=protocols/1/010f0194405aff49-a661a09f-0148-44f4-9fae-d9440c0c2932-000000/S6Gj-RsUAFiIU3szWSN6IFuo_zAJvTVIYqFPEEq4wMY=192).

*We thank the Editor for the suggestion. As previously stated in the first submission “The datasets generated and analysed during the current study and supplementary materials, and the components of the research methodology needed to reproduce the reported procedure(s) and analyses are publicly available on the Open Science Framework repository (DOI: 10.17605/OSF.IO/H7V5Y)”. We decided to share our data on the Open Science Framework repository as it is among the suggested repositories of PLOS ONE. In order to comply with the Editor’s request, we also shared our laboratory protocol in protocols.io at the following DOI: dx.doi.org/10.17504/protocols.io.yxmvm9m7nl3p/v1*

Journal Requirements:

1. When submitting your revision, we need you to address these additional requirements.

Please ensure that your manuscript meets PLOS ONE's style requirements, including those for file naming. The PLOS ONE style templates can be found at
[https://journals.plos.org/plosone/s/file?id=wjVg/PLOSOne_formatting_sample_main_body.pdf](https://track.editorialmanager.com/CL0/https:%2F%2Fjournals.plos.org%2Fplosone%2Fs%2Ffile%3Fid=wjVg%2FPLOSOne_formatting_sample_main_body.pdf/1/010f0194405aff49-a661a09f-0148-44f4-9fae-d9440c0c2932-000000/sD9_y6zfnrohEb7Na2fxgEt_OtmlNPXKychRBa53B0E=192) and
[https://journals.plos.org/plosone/s/file?id=ba62/PLOSOne_formatting_sample_title_authors_affiliations.pdf](https://track.editorialmanager.com/CL0/https:%2F%2Fjournals.plos.org%2Fplosone%2Fs%2Ffile%3Fid=ba62%2FPLOSOne_formatting_sample_title_authors_affiliations.pdf/1/010f0194405aff49-a661a09f-0148-44f4-9fae-d9440c0c2932-000000/_1bj2I4JmjfRliYPV_NtkZsV68Q6GvHqIjsrN16opqE=192)

*We edited the manuscript in order to meet PLOS ONE's formatting requirements.*

2. Thank you for stating the following financial disclosure:
[This work was supported by a European Research Council (ERC, [https://erc.europa.eu/homepage](https://track.editorialmanager.com/CL0/https:%2F%2Ferc.europa.eu%2Fhomepage/1/010f0194405aff49-a661a09f-0148-44f4-9fae-d9440c0c2932-000000/0_uquHW7vFpehinVFEkHI9ufeco1zPQL-A--zx4rExU=192)) Advanced Grant 2017, Embodied Honesty in real world and digital interactions (eHONESTY, Prot. 789058) (to S.M.A.) and Ministero dell'Università e della Ricerca (PRIN n. 20229JPNT7, [https://prin.mur.gov.it/](https://track.editorialmanager.com/CL0/https:%2F%2Fprin.mur.gov.it%2F/1/010f0194405aff49-a661a09f-0148-44f4-9fae-d9440c0c2932-000000/9ozdAhnMmjm0enGQPDQRQFzk-EF0j2iRwzL3Kq5xr0M=192)) (to S.M.A.)].

Please state what role the funders took in the study. If the funders had no role, please state: ""The funders had no role in study design, data collection and analysis, decision to publish, or preparation of the manuscript.""
If this statement is not correct you must amend it as needed.

Please include this amended Role of Funder statement in your cover letter; we will change the online submission form on your behalf.

*We included the Role of Funder statement in our cover letter as required. The cover letter now reads as follows: “This work was supported by a European Research Council (ERC, https://erc.europa.eu/homepage) Advanced Grant 2017, Embodied Honesty in real world and digital interactions (eHONESTY, Prot. 789058) (to S.M.A.) and Ministero dell'Università e della Ricerca (PRIN n. 20229JPNT7, https://prin.mur.gov.it/) (to S.M.A.). The funders had no role in study design, data collection and analysis, decision to publish, or preparation of the manuscript.”*

3. Thank you for uploading your study's underlying data set. Unfortunately, the repository you have noted in your Data Availability statement does not qualify as an acceptable data repository according to PLOS's standards.

At this time, please upload the minimal data set necessary to replicate your study's findings to a stable, public repository (such as figshare or Dryad) and provide us with the relevant URLs, DOIs, or accession numbers that may be used to access these data. For a list of recommended repositories and additional information on PLOS standards for data deposition, please see [https://journals.plos.org/plosone/s/recommended-repositories](https://track.editorialmanager.com/CL0/https:%2F%2Fjournals.plos.org%2Fplosone%2Fs%2Frecommended-repositories/1/010f0194405aff49-a661a09f-0148-44f4-9fae-d9440c0c2932-000000/On7qguM6zclPEsbgOhHrE1VPYQvNctcJjC8FsIZ6RTg=192).

*Although we already published our data in the Open Science Framework (included in your list of recommended repositories) at the following link: DOI: 10.17605/OSF.IO/H7V5Y, we also uploaded datasets and codes in Figshare at the following DOI: 10.6084/m9.figshare.28369424*


[Note: HTML markup is below. Please do not edit.]

5. Review Comments to the Author

Please use the space provided to explain your answers to the questions above. You may also include additional comments for the author, including concerns about dual publication, research ethics, or publication ethics. (Please upload your review as an attachment if it exceeds 20,000 characters)

Reviewer #1: Thank you for inviting me to review this paper. I love the focus – both in terms of an under researched condition, as well as the development of novel tests and the focus on signals beyond cardiac interoceptive accuracy. I really wanted to like this, but I have major concerns about the paper in its present form. These are detailed below, and I am on the fence between recommending rejection vs. major revision. On balance, I think that the authors should have the opportunity to improve their paper because this is a difficult and novel question, but there is a fair bit of work to be done.

Major:
1. Introduction - I think we should be careful to say that patients ‘misperceive’ signals given that the tasks used may have reporting biases (e.g., HCT, Water-load) and some studies in chronic pain show increased interoceptive accuracy (e.g., Todd et al., 2024; using the PAT).

*We thank the reviewer for their insightful suggestion. We agree that the term “misperceive” is not fully exhaustive for all the cases regarding chronic pain patients, even though the majority of the studies indicate a reduced ability to perceive internal bodily signals in this population. To address this, we have revised the text in the Introduction to replace the term "misperceive" and instead described interoceptive alterations or differences in interoceptive abilities between chronic pain patients and healthy controls. Moreover, we added the suggested reference (i.e. Todd et al., 2024), whose results are in contrast with the one we previously cited. We hope that this adjustment ensures that the language better reflects the complexity of interoceptive processes and the potential impact of task-specific characteristics. The sentence on line 37, page 3, now reads as follows: “Generally, individuals suffering from chronic pain and visceral hypersensitivity (i.e., increased sensitivity to pain originating from the internal organs) typically exhibit altered interoception, i.e. they perceive the physiological state of their body differently if compared to healthy controls (i.e. lower interoceptive accuracy [13–21] or higher interoceptive accuracy [Todd et al., 2024].”*

2. Introduction - The 2015 Garfinkel model is quite out of date now. Perhaps use the updated paper from those authors detailed below:
a. Suksasilp, C., & Garfinkel, S. N. (2022). Towards a comprehensive assessment of interoception in a multi-dimensional framework. Biological Psychology, 168, 108262.

*We thank the reviewer for their insightful suggestion. Initially, we decided to apply the Garfinkel et al. (2015) model to our project because, at the time of its conceptualisation, the Suksasilp and Garfinkel (2022) article had not yet been published. However, we agree on updating the interoception model to encompass all aspects currently considered in interoception field research. Therefore, we have revised the literature cited in the introduction, methods, and discussion sections of the manuscript, updating the terminology in line with the paper by Suksasilp and Garfinkel (2022).*

3. Introduction – we really shouldn’t be using the term interoceptive sensibility anymore given a wealth of evidence that different measures tap different processes (see references below). Please be more specific throughout the manuscript. I would also not rely on evidence from the HCT given the controversy about this task. I would like to see more nuance in the introduction.
a. Desmedt, O., Luminet, O., Walentynowicz, M., & Corneille, O. (2023). The new measures of interoceptive accuracy: a systematic review and assessment. Neuroscience & Biobehavioral Reviews, 105388.
b. Desmedt, O., Heeren, A., Corneille, O., & Luminet, O. (2022). What do measures of self-report interoception measure? Insights from a systematic review, latent factor analysis, and network approach. Biological Psychology, 169, 108289.

*We thank the reviewer for this comment and the suggested references. We appreciate the opportunity to refine the manuscript in light of recent evidence. First of all, we agree that the term "interoceptive sensibility" may no longer fully capture the complexity of processes measured by self-report tools. To address this issue, we revised the manuscript using more specific and up-to-date terminology, such as "self-reported interoceptive beliefs" or "subjective interoceptive beliefs" as in S*uksasilp & Garfinkel (2022)*. We also explicitly addressed the evolving understanding of interoceptive constructs in the introduction by adding the following sentence on line 51, page 3: “[...] (for an exhaustive description of all the different dimensions of interoception please refer to [22]) [...]”. Furthermore, we acknowledged that results based on questionnaires assessing subjective interoceptive beliefs should be interpreted with caution, due to the concerns on interoceptive questionnaires recently raised by Desmedt and colleagues (2022). For this reason, we edited the introduction at line 58, page 4, which now reads as follows: “[...]* Recent studies using subjective asses*sments have demonstrated that interoceptive self-regulation mediates the relationship between pain and depression in endometriosis patients, underscoring the crucial role of interoception in this condition [32].* *Nevertheless, Desmedt and colleagues (2022) suggest that uncertainty remains about whether current "interoceptive sensibility" and "self-report interoceptive scales" measures really assess a common construct. [...]”*

*Secondly, for what concerns the Heartbeat counting task (HCT), we acknowledge the ongoing debate about the validity of this task as a measure of interoceptive accuracy. To address this, we included a brief discussion in the introduction about the limitations of the HCT, citing relevant literature (e.g., Desmedt et al., 2023). Now the introduction on line 66, page 4 reads as follows: “[...] Patients and healthy controls performed the Heartbeat Counting Task to assess the cardiac domain [27], the Two-step Water Load Test for the gastric domain [33] and a novel version of a urinary interoceptive task designed to test bladder interoception. However, it is important to acknowledge the limitations of the Heartbeat Counting Task (HCT, [27]), which has been widely used to measure interoceptive cardiac accuracy. Recent evidence has raised concerns about the validity of the HCT, suggesting that it may not purely assess interoceptive accuracy but could also be influenced by other factors such as general cognitive abilities, beliefs about heart rate, and knowledge of cardiac rhythms. For instance, Desmedt et al. (2023) highlighted that the HCT results may reflect participants’ expectations or their ability to estimate time rather than their capacity to accurately perceive cardiac signals. These limitations underscore the importance of complementing this cardiac task with additional interoceptive tasks that assess other domains and provide a more comprehensive assessment of interoceptive abilities.[...]” We hope these revisions address the reviewer’s concerns and improve the clarity and rigour of our manuscript.*

4. Methods – 30 participants per group is tiny. Although a power analysis is included, there is no justification for the expected effect size of η2=0.14 which is VERY large. Perhaps this should be framed as a pilot study as the sample sizes are really too low to make firm conclusions. This will mean tempering of the quite bold conclusions in the Discussion as what is really needed is a well-powered replication of this effect, certainly not the development of interventions on the basis of this small study.

*We thank the reviewer for this comment. As mentioned by the reviewer, a power analysis was performed based on the power analysis and effect size indicated by Di Lernia et al., 2020 (*f = 0.4, α err prob. = 0.05, power = 0.80*) and Salamone et al., 2021 (* f = 0.40, α level of p = 0.05, and a power of 0.80*), both including interoception tasks and patients populations (Di Lernia et al., 2020 recruited chronic pain patients specifically). Moreover, studies regarding interoceptive abilities in chronic pain conditions usually recruit a comparable number of patients and controls: Solcà et al., 2020 (*24 chronic pain patients vs. 24 HC*); Martinez et al., 2018 (14 chronic pain patients vs. 13 HC); d'Alcalà, Webster & Esteves, 2015 (22 chronic pain patients vs. 37 HC); Borg et al., 2018 (21 fibromyalgia patients vs. 21 HC); Shizuma et al., 2021 (38 chronic pain vs. 35 HC); Valenzuela-Moguillansky, et al., 2017 (30 fibromyalgia patients vs. 29 HC). Thus, while we appreciate the reviewer’s suggestion to frame the study as a pilot, we would like to emphasize that the sample size in our study is consistent with that of several studies in the existing literature, which were not framed as pilot studies.*

*Nevertheless, we tried to soften the conclusions the reviewer is referring to, underlying the necessity of further studies to corroborate our results and suggesting the development of interventions as a further step forward. The edited discussion paragraph concerning the conclusions, on line 483, page 19, now reads as follows: “[...] This study introduces a non-invasive tool for assessing bladder interoception, which is topographically close to the uterus. On the one hand, identifying interoceptive markers in women with endometriosis may represent a potential aid for diagnosis. However, further studies are needed to corroborate these findings using different methodological approaches. On the other hand, the new urinary interoceptive task paves the way for the development of non-pharmacological therapeutic approaches aimed at improving patients' interoceptive abilities, such as the biofeedback approach relying on cardiac interoception [81]. Protocols combining biofeedback and interoceptive training could be designed in the future to improve interoceptive accuracy in women with endometriosis and shift their focus from pain to the perception of visceral signals. These proposed interventions remain speculative as the research on urinary interoceptive abilities is still in the early stages.”*

5. Methods – please provide a justification for this exclusion criteria “presence of endometriosis adhesions in the bladder area” – wouldn’t these be the people we would want to include?

*We thank the reviewer for the request for clarification. Indeed, the exclusion criterion “presence of endometriosis adhesions in the bladder area” seems to suggest that we excluded participants with adhesions in the pelvic region, not only specifically located on the bladder itself (e.g., adhesions on the ovaries, Fallopian tubes, pelvic peritoneum, uterosacral ligaments, or the external surface of the uterus). Nevertheless, we did not include in our sample women with endometriosis directly affecting the surface of the bladder, in order to rule out the possibility that impaired perception of bladder signals was due to an organic issue caused by adhesions on the organ being tested (i.e. the bladder itself). To avoid this confounding factor, we excluded women with bladder-specific adhesions and instead included those with adhesions in other pelvic areas. To further clarify this point, we revised the description of the exclusion criterion to specifically refer to “adhesions on the bladder” rather than the broader term “adhesions in the bladder area.” We hope this adjustment clarifies our approach.*

6. Methods - “chronic intake of drugs (except for those taken to treat endometriosis)” – please could you provide these details. For example, are more women on hormonal contraception in your endometriosis group than control group? Do you have differences in mental health between the groups? In the Discussion there is discussion of catastrophising and symptoms that very much seem to link to anxiety.

*We thank the reviewer for this comment. In general, the use of hormonal contraception was not an exclusion criterion during participant recruitment. Exclusion criteria considered medications that could have confounding effects on interoceptive tasks, for example, drugs known to influence the physiological processes assessed in the interoceptive tasks (e.g., medications affecting heart rate, bladder function, or gastric motility). Among the participants with endometriosis, some were on hormonal treatments as part of their clinical treatment of the condition. Specifically, 40% of patients were on progestin-based therapy, and 26.67% were on estrogen-containing regimens (as reported in Table 2). In contrast, we recognise that we did not systematically record its prevalence in the control group, as we did not consider it as a primary variable of interest. However, the absence of differences in interoceptive performance across subgroups with varying hormonal treatments within the endometriosis group (see the paragraph “Interoceptive Accuracy and Beliefs in Patients” in the Results section) may suggest this variable did not play a major role in affecting our results. We hope that this addresses the reviewer’s concern.*

*Concerning the differences between the two groups in terms of mental health, exclusion criteria included diagnosed neurological or psychiatric conditions. Nevertheless, we cannot exclude the possibility that patients with endometriosis, precisely because of the chronic nature of their symptoms, suffered from subclinical depression or anxiety. Thus, we included this limitation in the Discussion section on line 409, page 17, which now reads as follows: “In addition, it is important to consider the potential influence of co-occurring mental health disorders, on the observed differences in interoceptive accuracy between groups. Chronic health conditions like endometriosis are often associated with higher rates of anxiety and depression (Kigloo et al., 2024, Koller et al., 2023, van Barneveld et al., 2022), that may also affect interoceptive processing (Spinoni et al., 2024). Although our exclusion criteria included the presence of neurological or psychiatric conditions, we cannot rule out the possibility that patients with endometriosis suffered from subclinical depressive or anxious symptoms. Future studies should include questionnaires tackling mental health markers and, where possible, match groups on this variable to better disentangle the specific contributions of these factors to interoceptive accuracy.” We hope that by clarifying our recruitment methods, we answered the reviewer’s concerns.*

7. Methods – please could you include a justification for why no control tasks were included as part of the procedure to demonstrate no general perceptual issues that are non-interoceptive. This is a major limitation that needs to be acknowledged. For individuals who have chronic pain, this is particularly important.

*We thank the reviewer for pointing at this potential limitation. In this study, we chose not to include general control tasks aimed at ruling out non-interoceptive perceptual issues, as our primary focus was on exploring interoceptive abilities specifically within the cardiac, gastric, and urinary domains, which are particularly relevant to the symptomatology of endometriosis. This decision was guided by the hypothesis that interoceptive deficits in endometriosis are domain-specific and linked to altered visceral mechanisms associated with chronic pain in these patients. Moreover, given the already prolonged duration of the experimental protocol, adding an additional task (i.e. control task) would have been excessively time-demanding.*

*That said, we acknowledge that the absence of control tasks represents a methodological limitation of the study. Including such tasks would allow to confirm that the deficits observed in interoceptive accuracy are not influenced by general perceptual impairments unrelated to interoception. As highlighted by the reviewer, this limitation may be critical for individuals with chronic pain, as such conditions may involve broader perceptual or attentional alterations that could interact with interoceptive processing. That said, studies investigating interoceptive abilities in patients with chronic pain do not always include a control task to exclude other general perceptual impairments (see for example: Multi-somatoform disorder: Weiss et al., 2014; chronic pain: d'Alcalà et al., 2015; fibromyalgia: Rost et al., 2017; chronic neuropathic pain and chronic musculoskeletal pain: Di Lernia et al., 2020; complex regional pain syndrome: Solcà et al., 2020; chronic musculoskeletal pain: Shizuma et al., 2021). At any rate, we acknowledge this limitation in the discussion section, on line 404, page 16, which now reads as follows: "[…] Additionally, future studies would benefit from the integration of validated bladder and gastric symptom questionnaires, which could provide additional insights into the relationship between interoceptive accuracy and symptom burden. Another limitation of this study is the absence of control tasks to assess whether participants manifest non-interoceptive perceptual impairments. This is particularly relevant for individuals suffering from chronic pain, as they may exhibit broader perceptual or attentional deficits that could influence their interoception task performances. Hence, future studies should include control tasks that help isolate interoceptive-specific impairments from more generalised perceptual deficits.[…]”.*

8. Methods – I am not a fan of the HCT given the potential for reporting bias, nor the water load test given that the two-stage procedure may still be influenced by beliefs about stomach capacity and feelings of fullness. I am not entirely sure that the calculation of the urinary task actually provides the metric the authors are after. If you simply are looking for the consistency in minimum, would you not simply take the absolute difference between the two divided by total capacity. I am not sure why you have taken an average (e.g., divided by two). I would think this should be ABS(void 2 – void 3)/void 1.

*We thank the reviewer for the insightful comment that prompts us to clarify the rationale behind the calculation of our urinary interoception index. Our primary goal was not to assess the consistency between the two minimum stimuli (i.e., void2 and void3) but to assess participants’ ability to discriminate between the minimum stimulus (i.e., void2 and void3) and maximum stimulus (i.e., void1). The decision to have two measurements of minimum stimulus (i.e. void2 and void3) was exactly driven by the concern of having inconsistencies across measures, which would be properly captured by the reviewer’s suggested index. However, while the alternative formula effectively captures consistency, it does not provide a direct measure of how participants differentiate between the minimum and maximum bladder sensations. To illustrate, let’s consider the following scenario: Subject 1 with void1 = 500 ml, void2 = 70 ml, and void3 = 70 ml, and Subject 2 with void1 = 500 ml, void2 = 400 ml, and void3 = 400 ml. Using our formula [1-((void2-void3)/2)/void1], Subject 1, would show a higher ability to discriminate between minimum and maximum stimuli, having an index of 0.86, whereas Subject 2, whose minimum voids are much closer to void1, would result less accurate in discriminating between the two different stimuli, having an index of 0.20. In contrast, using the formula proposed by the reviewer [*ABS(void 2 – void 3)/void 1*], both participants would have the same index (0), despite their differing abilities to differentiate between bladder sensations. This example highlights that while the reviewer’s approach is a valuable measure of consistency, it would not fully capture the interoceptive accuracy that our task aims to assess. Moreover, our response to Comment 9 of the reviewer confirms that there is no inconsistency between void2 and void3 in either the control group (W=300, p=0.171) or the patient group (W=265, p=0.510). Thus, we believe that the index we conceived provides the metric we were looking for.*

9. Methods – out of interest, were void 2 or void 3 consistently greater or larger than each other in either group?

*We thank the reviewer for the question. We can confirm that void2 and void3 were not consistently different neither in the control group (W=300, p=0.171) nor in the patient’s group (W=265, p=0.510). We added this analysis to the supplementary materials as we think it completes our results. The supplementary material “Further analyses” paragraph now reads as follows: “[...] We also performed two non-parametric t-tests to confirm that void2 and void3 were not consistently different from each other in either group. Results showed that void2 did not significantly differ from void3 neither in the control group (W=300, p=0.171) nor in the patients' group (W=265, p=0.510).”*

10. Methods – could you provide a rationale for why you didn’t include a validated bladder symptom or gastric symptom questionnaire?

*We thank the reviewer for this insightful comment. We acknowledge the value of validated bladder and gastric symptom questionnaires in studies assessing interoception and related measures. In this study, our primary aim was to focus on interoceptive accuracy, measured through task performance across the cardiac, gastric, and urinary domains, rather than on self-reported gastric or bladder symptom severity or prevalence. The decision not to include validated symptom questionnaires was based on the rationale that such measures primarily assess subjective symptomatology (e.g., pain severity, frequency of bladder or gastric disturbances) rather than the ability to detect internal physiological signals, which was the central focus of our research. Thus, we decided to include an already validated questionnaire for interoceptive gastric sensations (Van Dyck et al., 2016, see tables S1 and S2) and to create a bladder interoceptive questionnaire because, to the best of our knowledge, the existing questionnaires assessing bladder sensations (e.g. bladder diaries: Naoemova et al., 2008; Urgency Perception Scale: Cardozo et al., 2005; Urgency Perception Score: Blaivas et al., 2007; for a complete review see MacDiarmid, 2009) are customised for patients who suffer from bladder disorders and primarily focus on the sense of urgency. However, we agree that incorporating validated symptom questionnaires could provide valuable additional insights, particularly when examining the relationship between symptom burden and interoceptive performance. Thus, we added this point in the revised Discussion section. We hope this explanation clarifies our approach and addresses the reviewer’s concern. The edited discussion section on line 401, page 16, now reads as follows: “[...]Concerning the urinary interoceptive task, its primary drawback lies in its time-consuming nature, an issue that should be addressed in future studies of urinary interoception. Additionally, future studies would benefit from integrating validated bladder and gastric symptom questionnaires, which could provide additional insights into the relationship between interoceptive accuracy and symptom burden.[...]”*

11. Data analysis – the sample is far too small to run a factor analysis unless this has been validated elsewhere and should not be used. I would suggest looking at individual items and correcting for comparison.

*We thank the reviewer for the valuable feedback regarding the factor analysis and the sample size. We acknowledge that our sample may be slightly small to conduct a robust factor analysis (i.e., Bartlett’s test of sphericity is significant (X^2^ =85.5, df=10, p <0.001), which means that the variables are sufficiently correlated to justify a factor analysis, and the Kaiser-Meyer-Olkin (KMO) is 0.579, which means that the sample adequacy is borderline, but factor analysis is still feasible with caution). Nevertheless, we understand the reviewer’s concern, thus we revised our analysis by focusing on individual items rather than relying on a factor structure.*

*To account for multiple comparisons, we applied the FDR correction using R studio software to our p-values (Benjamini & Hochberg, 1995). The adjusted p-values are as follows: Urinary attention: 0.0449; Urinary interference: 0.0212; Urinary worry: 0.0071; Urinary night-time frequency: 0.0449; Urinary day-time frequency: 0.0317.*

*These results indicate that significant effects remain even after controlling for multiple comparisons. We revised the manuscript as follows:*

*Data analyses on line 225, page 10: “As for the five questions assessing participants’ bladder ~~sensibility~~ beliefs, we first ran a series of Wilcoxon rank-sum tests on each question assessing attitude towards bladder stimuli between the control and patient groups, controlling for multiple comparisons with False Discovery Rate (FDR) correction.”*

*Results on line 285, page 12: “To assess differences in bladder interoceptive beliefs, we performed a series of Wilcoxon rank-sum tests on each question assessing attitude towards bladder stimuli between the control and patient groups. To control for multiple comparisons, we performed the False Discovery Rate (FDR) correction. The adjusted p-values are as follows: Urinary attention: 0.0449; Urinary interference: 0.0212; Urinary worry: 0.0071; Urinary night-time frequency: 0.0449; Urinary day-time frequency: 0.0317. A significant difference was found for urinary attention (W = 317, p = 0.0449, FDR-corrected), urinary interference (W = 273, p = 0.0085, FDR-corrected), urinary worry (W = 237.5, p = 0.0014, FDR-corrected), urinary nighttime (W = 318.5, p = 0.0427, FDR-corrected), and urinary daytime (W = 293, p = 0.0190, FDR-corrected).”*

*Discussion on line 431, page 17: “Crucially, when looking specifically at urinary interoceptive beliefs (measured using the questions developed in this study), patients appear to focus more on bladder signals, to experience greater interference on daily activities due to bladder stimuli, express greater concern about them, and experience more night and day time need to empty the bladder compared to controls.”*

*Moreover, we deleted the supplementary materials paragraph concerning the factor analysis and changed Figure 5, reporting all the t-tests we ran for each question assessing participants’ attitudes towards bladder stimuli. The new Figure 5, with its caption, is pasted below.*

***Fig.5. Bladder Interoceptive Beliefs.*** *Non-parametric t-tests to explore differences in a) attention to bladder stimuli between patients with endometriosis and healthy controls (higher scores indicate greater attention towards bladder stimuli); b) interference of bladder stimuli with daily activities (higher scores indicate greater interference of bladder stimuli); c) concern towards bladder stimuli between patients with endometriosis and healthy controls (higher scores indicate greater concern towards bladder stimuli); d) night-time need to empty the bladder (higher scores indicate higher need to empty the bladder during nighttime); e) day-time need to empty the bladder (higher scores indicate higher need to empty the bladder during daytime).*

12. Results – as your correlation between tasks is driven by the healthy controls, could you please colour code groups in figure 3 and add the results from the supplement in the figure legend. Also, in figure legends please include the direction of scores – e.g., high score = better perception.

*We thank the reviewer for this comment. Following the suggestions, we colour-coded the groups in Figure 3 (see below) and added the result from supplementary materials in the figure legend. We also made explicit the direction of the scores in all the figure legends. We hope that after these changes, the figures and their legends will be more straightforward.*

*The edited legend captions, on pages 11, 12 and 13 now reads as follows:*

***“Fig 3. Correlation between the Two-step Water Load and the Urinary Interoceptive Task.*** *The higher the participants’ gastric interoceptive accuracy (i.e., higher scores indicate higher ability to perceive gastric signals), the higher their urinary interoceptive accuracy (i.e., higher scores indicate higher ability to perceive urinary stimuli). Running correlation analyses separately for each group (healthy controls vs. patients with endometriosis) revealed that the novel urinary task did not correlate with the Heartbeat Counting Task (R=0.2389, p=0.2034) but did correlate with the Water Load Test (R=0.5522, p=0.001557) in healthy controls. In contrast, in the endometriosis group, there were no significant correlations with either the Heartbeat Counting Task (R=0.1516, p=0.4239) or the Water Load Test (R=0.1357, p=0.4745).*

***Fig. 4. Interoceptive Accuracy Tasks.*** *T-test to explore differences in the a) cardiac interoceptive task between patients with endometriosis and healthy controls; b) gastric interoceptive task between patients with endometriosis and healthy controls; c) urinary interoceptive task. Higher scores indicate a greater ability to detect interoceptive signals (i.e., cardiac, gastric and urinary). Significance: ‘***’ p≤.001; ‘n.s.’ non-significant results.*

**Fig.5. Bladder Interoceptive Beliefs.** Non- parametric t-tests to explore differences in a) attention to bladder stimuli between patients with endometriosis and healthy controls (higher scores indicate greater attention towards bladder stimuli); b) interference of bladder stimuli with daily activities (higher scores indicate greater interference of bladder stimuli); c) concern towards bladder stimuli between patients with endometriosis and healthy controls (higher scores indicate greater concern towards bladder stimuli); d) night-time need to empty the bladder (higher scores indicate higher need to empty the bladder during nighttime); e) day-time need to empty the bladder (higher scores indicate higher need to empty the bladder during daytime).

13. Results – I think that given so many analyses, I would not discuss marginally significant results. As you have a lot of questionnaires/analyses, where these are answering the same question, I think it would be good to include a correction for all of these comparisons. Not being too strict, but maybe p=.025 should be the alpha here as there are many comparisons. At a minimum, you should detail which survive correction for multiple comparisons where the same family of tests answer the same question (e.g., if looking at patients vs. controls on interoceptive accuracy tasks, you should correct for the three comparisons).

*We thank the reviewer for the thoughtful feedback regarding multiple comparisons and the interpretation of marginally significant results. We understand the concern about the number of analyses conducted and appreciate the suggestion to apply a more stringent alpha threshold.*

*However, we conceived interoception as a domain-specific construct, where different bodily systems contribute to interoceptive processing in distinct ways. Although the three interoceptive accuracy tasks all assess interoceptive abilities, they target different body domains (cardiac, gastric, and urinary), each with its own physiological and perceptual characteristics. As such, we believe it is important to consider them as independent measures, rather than applying corrections across them as if they were part of the same family of tests. This approach aligns with previous literature (e.g., Ferentzi et al., 2018; Desmedt et al., 2023; Garfinkel et al., 2016; Whitehead & Drescher, 1980; Herbert et al., 2012; Füstös et al., 2013; Garfinkel et al., 2017), which treats interoceptive accuracy across different bodily domains as distinct constructs rather than a single unified measure.*

*Similarly, the various self-report questionnaires included in our study assess different psychological dimensions related to interoception, rather than overlapping constructs. While we acknowledge the importance of controlling for multiple comparisons in cases where tests address the same research question, we believe that applying corrections across interoceptive domains or across conceptually distinct questionnaires would not be appropriate in this context.*

*That said, for the sake of clarity, we adjusted the p-value using a Bonferroni correction (corrected α= 0.016). After adjustment, the results for the gastric (WLT) and urinary (UIT) water load tests remained significant, while the heartbeat counting task (HCT) remained non-significant. Thus, we believe that applying this correction in the manuscript would not be conceptually justified, as it assumes that interoceptive accuracy is a single construct rather than being body-region specific.*

14. Figures – have come out a bit blurry. Could you try making it easier to see the words in the figures.

*We thank the reviewer for pointing this out. We made the words in the figures bigger and increased the quality of the figures. We hope that now they are easier to read.*

15. Discussion – when discussing the results of the gastric and urinary task relationship, it would be nice to also acknowledge that tasks that share similar formats/demands are often related and discuss this with a view to studies that don’t often report relationships across domains. See reference below for example.
a. Whitehead, W. E., & Drescher, V. M. (1980). Perception of gastric contractions and self‐control of gastric motility. Psychophysiology, 17(6), 552-558.
b. Ferentzi, E., Bogdány, T., Szabolcs, Z., Csala, B., Horváth, Á., & Köteles, F. (2018). Multichannel investigation of interoception: Sensitivity is not a generalizable feature. Frontiers in human neuroscience, 12, 223.

*We thank the reviewer for the suggestion to improve our manuscript. We added a paragraph discussing the literature's existing debate on whether a relationship in interoceptive abilities across different body domains exists. As suggested, we also acknowledged the possibility that urinary and gastric tasks are related because of their similar format. The discussion paragraph on line 350, page 15, now reads as follows: “[...] These findings contribute to a broader debate within the literature on whether interoceptive abilities correlate across different body domains or remain modality-specific. For instance, Ferentzi et al. (2018) proposed that interoceptive sensitivity is primarily a modality-specific feature, suggesting that individuals’ accuracy in detecting internal signals is confined to specific bodily systems and does not extend to others (see Desmedt et al., 2023; Garfinkel et al., 2016; for other results supporting the modality-specific perspective). This perspective is supported by our observation that cardiac interoception, as measured by the Heartbeat Counting Task, did not correlate with either gastric or urinary interoception. In contrast, Whitehead and Drescher (1980) argued for a more generalised tendency to be aware of or attend to visceral events, suggesting that individuals sensitive to one type of internal signal may also be sensitive to others (see Herbert et al., 2012; Füstös et al., 2013; Garfinkel et al., 2017; for other results supporting the generalised interoceptive ability perspective). This view aligns with our results showing a positive correlation between gastric and urinary interoception. Thus, our findings sit at a midway point in this debate, suggesting that interoceptive abilities may generalise within certain domains (e.g., visceral systems like the stomach and bladder) but remain distinct across others (e.g., cardiac versus non-cardiac domains). This may reflect the intrinsic difference between the visceral signals tackled in the three tasks. Indeed, heart signals at rest are less salient than those coming from the stomach and bladder. In other words, when not engaged in a specific task, individuals may not consistently prioritise signals originating from the cardiac region, whereas they tend to focus more on gastric and bladder signals due to their role in signalling urgent homeostatic and allostatic needs. Moreover, stomach and bladder signals share a neural pathway, as both gastric and bladder motility (i.e., the stomach contraction leading to stomach emptying and bladder contraction leading to urination) are controlled by an increased phasic activity of the parasympathetic pathways whereas the increase of the heart rate is guided by a higher phasic activity of the sympathetic pathway [50]. Another possibility is that the results of the gastric and urinary interoception accuracy tasks align in the same direction due to the intrinsic similarities in the nature of these tasks. Indeed, both tasks rely on visceral feedback during water-loading protocols, which may lead to overlapping cognitive and physiological processes. Additionally, recent studies show that tasks that share similar formats or instructions tend to show relationships, as participants may approach them with comparable strategies or mental frameworks [Suksasilp, C., & Garfinkel, S. N. (2022)]. [...]”*

16. Discussion – not sure if you should recommend the Whitehead et al., (1977) procedure. There is a wealth of data on the issues with this task. See the review below.
a. Brener, J., & Ring, C. (2016). Towards a psychophysics of interoceptive processes: the measurement of heartbeat detection. Philosophical Transactions of the Royal Society B: Biological Sciences, 371(1708), 20160015.

*We thank the reviewer for highlighting concerns about the Whitehead et al. (1977) procedure and recommending Brener and Ring (2016). As noted, the Heartbeat Counting Task (HCT) has limitations, including its reliance on subjective self-report and susceptibility to cognitive biases. We agree with Brener and Ring (2016) that the Heartbeat Discrimination Task version by Brener & Kluvitse (1988) is a more robust alternative, as it objectively assesses the synchrony between external tones and heartbeats, reducing potential confounds. Thus, we decided to change the reference from Whitehead et al. (1977) to Brener & Kluvitse (1988) and we thank the reviewer for giving us the possibility to strengthen our manuscript.*

17. Discussion – somewhere you should outline the issues with the other two tasks not just the HCT, including that they aren’t exactly measuring the same thing despite being correlated (fullness vs consistency) as well as discuss their limitations.

*We thank the reviewer for this valuable feedback. We appreciate the suggestion to expand on the limitations of all three interoceptive tasks. In our manuscript, we already noted that the urinary interoceptive task (UIT) is time-consuming. Moreover, we wish to clarify that it is true that despite being correlated, the two tasks do not measure the same thing. Nevertheless, the gastric water load index does not measure participants’ fullness itself, but rather, the efficiency of gastric interoception refers to participants’ ability to perceive their own fullness in relation to the actual volume of water consumed. Likewise, the urinary interoceptive task does not only aim to measure participants’ consistency in perceiving their minimum stimulus but, as its primary aim, it was conceived for assessing participants’ ability to discriminate between two different bladder stimuli (i.e., the minimum vs. maximum). We agree with the reviewer's suggestion to discuss both urinary and gastric interoceptive tasks’ limitations. Thus, we added a paragraph in the discussion, on line 392, page 16, which now reads as follows: Similarly, despite being correlated, the gastric and urinary interoceptive tasks assess two different constructs (i.e., participants’ ability to perceive their stomach fullness in relation to the actual volume of water consumed the former, and participants’ ability to discriminate between two different bladder stimuli the latter). Moreover, both tasks have limitations that need to be acknowledged. The principal drawback of the gastric water load task is that it relies on self-reported fullness, that is subjective and might be influenced by cognitive biases, mood, and prior experiences (Van Dyck et al., 2016; Ahlich et al., 2023). Moreover, since the test assesses fullness using water rather than solid food, its ecological validity is limited, making it less reflective of real-world eating behaviours. Lastly, individuals may interpret "maximum fullness" differently, leading to variability in results (Van Dyck et al., 2016). Concerning the urinary interoceptive task, its primary drawback lies in its time-consuming nature, an issue that should be addressed in future studies of urinary interoception. [...]”.*

18. Discussion – I think that in discussion of the HCT, one should be careful to describe not just general limitations, but how this would have influenced YOUR results. E.g., perhaps patients compensate for poor perception using heart rate knowledge which they may be expected to have more of, given they may monitor their bodily signals more. Some evidence to this effect here for anxiety:
a. Biotti, F., Barker, M., Carr, L., Pickard, H., Brewer, R., & Murphy, J. The effects of the SARS-CoV-2 pandemic on self-reported. PloS one.

*We thank the reviewer for this insightful comment. We agree that, beyond discussing the general limitations of the Heartbeat Counting Task (HCT), it is important to consider how these limitations may have specifically influenced our results. To address this, we expanded the discussion to acknowledge the possibility that patients with endometriosis, due to their chronic pain condition and increased attention to bodily sensations, might compensate for poor interoceptive accuracy by relying on heart rate knowledge.*

*We have revised the Discussion section accordingly and appreciate the valuable reference suggestion. The discussion paragraph on the limitations of the Heartbeat counting task, on line 383, page 16, now reads as follows: “[...] pressure [56]. Undoubtedly, these limitations of the HCT may have influenced our results. In particular, since individuals with chronic pain conditions are used to scanning their bodies for painful sensations, it is possible that patients with endometriosis compensate for reduced interoceptive accuracy by relying on knowledge of their heart rate, rather than directly perceiving their cardiac signals. This aligns with previous research showing that individuals with anxiety, who also tend to monitor bodily signals closely, may use cognitive strategies rather than true interoceptive perception in heartbeat detection tasks (Biotti et al., 2023). Future studies could complement the HCT with additional cardiac interoceptive measures, such as the Heartbeat Discrimination Task, to better disentangle perceptual accuracy from cognitive estimation strategies. [...]”.*

19. Discussion – relatedly, as chronic health conditions are often associated with poor mental health, one should also acknowledge that differences may not be driven by the condition, but may be driven by other co-occurring conditions as presumably groups were not matched for this.

*We thank the reviewer for pointing out the potential role of mental health conditions in influencing the observed differences. As already stated in a previous response to the reviewer, although the exclusion criteria included the presence of neurological or psychiatric conditions (such as anxiety, and depression), we cannot exclude the possibility that patients with endometriosis, precisely because of the chronic nature of their symptoms, suffered from subclinical depressive or anxious symptoms. We acknowledge that chronic health conditions often co-occur with mental health challenges, which may contribute to differences between groups. Thus, we included this limitation in the Discussion section on line 409, page 17, which now reads as follows: “[...] In addition, it is important to consider the potential influence of co-occurring mental health disorders, on the observed differences in interoceptive accuracy between groups. Chronic health conditions like endometriosis are often associated with higher rates of anxiety and depression (Kigloo et al., 2024, Koller et al., 2023, van Barneveld et al., 2022) , that may also affect interoceptive processing (Spinoni et al., 2024). Although our exclusion criteria included the presence of neurological or psychiatric conditions, we cannot rule out the possibility that patients with endometriosis suffered from subclinical depressive or anxious symptoms. Future studies should include questionnaires tackling mental health markers and, where possible, match groups on this variable to better disentangle the specific contributions of these factors to interoceptive accuracy.[...]”*

20. Discussion – I do not agree that these results suggest a distinction between objective vs. self-report, I think you should be careful given there are self-reported differences when you look at bladder symptoms, specifically. As such, I would acknowledge here the possibility that general measures may not capture specific differences that relate to a single bodily domain.

*We thank the reviewer for pointing at this difference. In our discussion, we highlight the different directions between the results of interoceptive tasks and the answers to interoceptive beliefs questionnaires as, in line with the literature, we find differences between these two interoceptive components. Nevertheless, we agree with the reviewer in saying that, in a domain-specific perspective of interoceptive abilities, it is possible that generic questionnaires assessing interoceptive beliefs, such as MAIA-II and BPQ, are not able to tackle the differences related to a single body domain. Hence, we acknowledge the limitation of these general interoceptive beliefs measures, suggesting that future studies should include domain-specific questionnaires for interoceptive beliefs, in order to better disentangle the possible difference between participants’ interoceptive abilities measured through interoceptive accuracy tasks and participants’ interoceptive beliefs. We edited the discussion at line 424, page 17, which now reads as follows: “[...] Considering Suksasilp & Garfinkel’s multi-dimensional interoceptive framework [22] and in line with recent literature [57], our data highlight the complexity of interoceptive processing in women with endometriosis. In particular, although when looking at MAIA-II and BPQ questionnaires patients with endometriosis appear comparable with controls in their subjective propensity to focus on internal body signals, they are ultimately less accurate than controls in discriminating urinary and gastric signals. Crucially, when looking specifically at urinary interoceptive beliefs (measured using the questions developed in this study), patients appear to focus more on bladder signals and express greater concern about them than controls. These findings shed light on a potential limitation of interoceptive questionnaires like the MAIA-II and BPQ in assessing interoceptive beliefs, as they may fail to capture nuanced differences related to specific bodily domains. Thus, future studies could introduce more tailored tools capable of providing a precise evaluation of domain-specific interoceptive beliefs, particularly for patient populations with conditions like endometriosis that affect specific organs and body regions. Moreover, we speculate[...]”*

21. Discussion – the paragraph related to neural underpinnings is unnecessary and can be removed.

*We thank the reviewer for the suggestion. We agreed with the reviewer by considering the paragraph specifically referring to interoceptive neural underpinning unnecessary, thus we decided to delete it, together with the related reference “63”. Nevertheless, we chose to keep the paragraph relative to the brain salience network as we think it is essential to link interoception with chronic pain. We decided to summarise this paragraph in order not to make it out of context. The paragraph, beginning on line 447, page 18 now reads as follows: “Our study also aimed to compare pain perception between patients with endometriosis and healthy women and the possible relationship between chronic pain and interoception. The interoceptive system, crucial for maintaining homeostatic control, integrates internal and external information to predict and manage bodily state changes [15,16,28,64,65] and it is tied to the Salience of an interoceptive stimulus, which depends upon its importance for bodily homeostasis [66]. Importantly, the brain salience network seems connected on several levels to the interoceptive cortex, which is crucial in predicting the interoceptive salience of a stimulus [67,68]. Thus, pain experience may derive from a predictive process involving top-down signals about the homeostatic significance of nociceptive inputs [28,69]. Another theory supporting the link between the interoceptive cortex and the salience network relies on the concept of Responsivity [70], according to which the brain states are influenced by the dynamic integration of exteroceptive stimuli and interoceptive signals [28,70]. In line with this perspective, abnormal interpretations of interoceptive signal salience may lead to unpredictable responsivity in patients with chronic pain [70], ultimately leading to increased pain tolerance as a strategy for suppressing unnecessary inputs within an already overwhelmed system [21,28].”*

22. Discussion – I would not discuss the result that was not significant in as much detail.

*We thank the reviewer, and we tried to avoid describing the non-results in detail, mainly focusing on future perspectives. The discussion paragraph concerning the non-significant result on line 470, page 19, now reads as follows: “[...] Furthermore, results show that women with endometriosis reported experiencing higher pelvic pain, pain during menstruation, sexual intercourse, and defecation than controls. The moderating effect of the group on the relationship between interoception and chronic pain is not large enough to draw firm conclusions. In order to better understand the relationship between interoception and pain in endometriosis, future studies should include a measure assessing objective pain[...]”*

Minor:
1. Abstract – should be cardiac interoception (or interoceptive accuracy) tasks.

*We thank the reviewer for spotting the inaccuracy. We edited the sentence in the abstract which now reads as follows: “[…] studies have explored interoceptive deficits in chronic pain individuals mainly using questionnaires or cardiac interoceptive accuracy tasks […].”*

1. Introduction - Is the term interstitial cystitis still in use? I thought it was now called chronic urinary tract infections.

*We thank the reviewer for the thoughtful question. The term "interstitial cystitis" (IC) is still used in medical practice and research. It specifically refers to a chronic condition characterised by bladder pain, urinary urgency, and frequency, without evidence of a clear bacterial infection. While some overlap in symptoms can occur, it is distinct from chronic urinary tract infections (UTIs), which are recurrent infections caused by bacterial pathogens. The distinction is important because the diagnostic criteria and management strategies differ significantly between these two conditions. We hope this clarification is helpful, and we appreciate the reviewer’s interest in this topic.*

2. Introduction – please use consistent terminology throughout (e.g., domains, different bodily districts etc).

*We thank the reviewer for the valuable feedback regarding the terminology in the Introduction. We acknowledge the importance of maintaining consistency throughout the manuscript. To address this, we consistently used the term "body domain(s)" throughout the text. All instances of alternative terms, such as "bodily districts," have been revised accordingly to ensure clarity and coherence.*

--------------------------------------------------

Reviewer #2: The study compared interoceptive accuracy and interoceptive beliefs in women with endometriosis and healthy controls across cardiac, gastric, and urinary domains. Women with endometriosis showed reduced accuracy in gastric and urinary interoception but not cardiac interoception. Despite this, they reported heightened bladder interoceptive beliefs, characterised by increased attention and concern about bladder signals. Group marginally moderated the effect of chronic pain on urinary interoception, suggesting that heightened pain sensitivity may impair interoceptive accuracy. The novel Urinary Interoceptive Task correlated with gastric but not cardiac interoceptive accuracy, highlighting shared neural pathways for gastric and bladder signals.
This study highlights dissociations between objective interoceptive accuracy and subjective interoceptive beliefs in women with endometriosis, suggesting that interoceptive impairments may result from pain hypersensitivity and hypervigilance. The findings provide valuable insights for developing non-invasive diagnostic tools and interoceptive training therapies for chronic pain management. This study addresses a relevant topic in interoception and employed a well-designed method. The results are informative and important in further elucidating the relationship between chronic pain and interoception. The manuscript offers an interesting read and would make a meaningful contribution to the interoception and pain literature following revision and clarification of the following details:

Introduction
The terminology around interoceptive dimensions would benefit from updating to match the more recent multidimensional framework by Suksasilp & Garfinkel (2022), Towards a comprehensive assessment of interoception in a multi-dimensional framework, and change terms such as interoceptive sensibility to interoceptive beliefs, and interoceptive awareness to interoceptive insight, accordingly.

*We thank the reviewer for the insightful suggestion. Initially, we decided to apply the Garfinkel et al. (2015) model to our project because, at the time of its conceptualisation, the Suksasilp and Garfinkel (2022) article was not published. However, we agree on the necessity to update the interoception model to encompass all the new aspects currently considered in interoception field research. Therefore, we have revised the literature cited in the introduction, methods, and discussion sections of the manuscript, updating the terminology in line with the paper by Suksasilp and Garfinkel (2022). As suggested by the reviewer, “interoceptive sensibility” now reads as “interoceptive beliefs” and “interoceptive awareness” reads as “interoceptive insight”. We also made sure to update the definitions of the different dimensions of interoception following Suksasilp and Garfinkel’s (2022) multi-dimensional interoceptive framework.*

Method
POWER analysis: More detail is required regarding the test used. Please clarify which test family, statistical test, type of power analysis, and other relevant input parameters (e.g. number of tails) were entered.

*We thank the reviewer for this comment. As required, we specified all the parameters used to perform the power analysis. The method paragraph concerning the power analysis, on line 124, page 7, now reads as follows: “An a priori Power Analysis, based on previous literature [Di Lernia et al., 2020; Salamone et al., 2021], was carried out to determine the sample size using the G*Power 3.1.9.4 software [43], setting the following parameters: test family= F tests; statistical test= ANOVA fixed effect, one-way; effect size f= 0.4 (η2=0.14); Power=0.80; α err prob.=.05; number of groups=2; Critical F = 4.03. The Power analysis suggested recruiting 26 individuals per group.”*

Water load test:
How long apart were the two drinking phases; i.e., did drinking phase 2 follow phase 1 immediately?
State the meaning of the index regarding interoceptive accuracy (higher index, higher accuracy?)

*We thank the reviewer for the questions. We confirm that the drinking phase 2 immediately followed the drinking phase 1. We included this clarification in the revised version of the manuscript. Moreover, we specified the meaning of the gastric interoceptive index as required. The paragraph on line 160, page 8, now reads as follows: “[…] in the second phase, immediately after the first one and lasting a maximum of five minutes, participants were asked to drink water until they reached the point of maximum gastric fullness (Δfull_ml). At the end of the two phases, the ratio between the volumes of water ingested in the two phases is calculated to obtain an individual gastric interoception index (sat_%=(sat_ml/total_ml)*100). The index obtained is a number between 0 and 100, dimensionless and therefore independent of the participant's stomach volume: the higher the index, the higher the subject's gastric interoceptive accuracy.[…]”.*

Urinary interoception task:
On page 7, lines 172–174, you mention the use of a VAS for participants to rate their bladder sensations. Could you clarify in the manuscript which specific rating on the scale (0–10) was defined as the 'minimum urinary stimulus'?

To enhance the reliability and validity of the findings, please clarify whether the same subjective rating of the 'minimum urinary stimulus' was consistently applied within participants across the void2ml and void3ml conditions. For example, if a participant indicated '4' as the minimum in the void2ml condition, was the same rating ('4') considered as the minimum in the void3ml condition?

If consistency was not maintained, I recommend noting this as a limitation in the manuscript, given the importance of this measure in calculating the Urinary Interoceptive Index. Specifically, discuss how variability in subjective threshold s and baseline urinary sensations might have influenced condition comparability (e.g., void2ml vs. void3ml within participants), introduced noise into the data, or affected the interpretation of findings.

*We thank the reviewer for the valuable feedback. To clarify, the 'minimum urinary stimulus' was consistently defined in the manuscript (page 7, lines 172–174) as the first desire to void when participants perceived a slight sensation to urinate and considered that "I could go, but it’s not necessary," with ratings captured on a VAS scale from 0 (no bladder stimulus) to 10 (maximum urinary stimulus). Although a specific VAS score was not predefined as a minimum stimulus (e.g., for a participant the minimum stimulus could have been ‘1’ while for another ‘3’), participants were instructed to consistently apply the same subjective criterion in the void2ml and void3ml conditions. Consistency was further ensured by comparing the volumes of urine expelled during these conditions, and the lack of significant discrepancies confirmed that participants reliably interpreted and applied the threshold across both conditions. To corroborate the lack of discrepancies between the two minimum voids in each group, we added the following paragraph to the supplementary material “Further analyses” paragraph: “We also performed two non-parametric t-tests to confirm that void2 and void3 were not consistently different from each other in either group. Results showed that void2 did not significantly differ from void3 neither in the control group (W=300, p=0.171) nor in the patients' group (W=265, p=0.510).” This design strengthens the reliability of the Urinary Interoceptive Index by minimizing variability in subjective thresholds, ensuring condition comparability, and reducing noise in the data. As requested, we clarified this aspect in the manuscript section, explaining the urinary interoceptive task. The paragraph in the methods section on line 191, page 9, now reads as follows: “Each time, before drinking and upon reaching the maximum and minimum stimuli, participants were asked to rate their bladder sensations with a VAS from 0 (indicating no bladder stimulus at all) to 10 (indicating the maximum urinary stimulus). Although a specific VAS score was not predefined as a minimum stimulus (i.e., each participant could indicate the desired number as minimum stimulus, following their own sensations), participants were instructed to consistently apply the same subjective criterion in both the void2ml and void3ml conditions (i.e., for each participant the VAS score assigned to void2ml as minimum stimulus should have been the same in void3ml). ”*

Line 183: Replace "her" with "their" to ensure inclusive language.

*We thank the reviewer for the suggestion. We replaced “her” with “their” as required to ensure inclusive language. The sentence, now on line 205 page 10, reads as follows: “The bigger this ratio, the better the subjects are at discriminating between two different stimuli coming from their bladder”.*
