# Supplementary material for: Impaired gastric and urinary but preserved cardiac interoception in women with endometriosis
Source: PLoS One. 2025 May 5;20(5):e0322865. doi: 10.1371/journal.pone.0322865 (PMC12052146; doi:10.1371/journal.pone.0322865)
Supplement: Attachment — Submitted filename: Respose to reviewers.docx [file pone.0322865.s005.docx]

*We thank the Editor for giving us the chance to further improve our manuscript by addressing reviewers’ comments. Please find below a point-by-point reply to the remarks of the reviewers with changes to the original manuscript made after the first rebuttal highlighted in yellow and the changes made after the second rebuttal highlighted in light blue. We hope that you and the reviewers are happy with the revised manuscript and that it can be accepted for publication.*

Review Comments to the Author

Reviewer #1: Thank you to the authors for their efforts in responding to comments. This is mostly really well achieved. I only have a few remaining concerns.
1. Bladder endometriosis exclusion and the issue of hormonal contraception – I would like to see those added as a limitation alongside the issue of mild/moderate mental health symptoms, please.

*We thank the reviewer for allowing us to further improve our manuscript. We edited the limitation section of the discussion by following the reviewer’s suggestion. The paragraph at line 432, page 17, now reads as follows: “**Concerning inclusion and exclusion criteria, patients with endometriosis adherences directly affecting the surface of the bladder were excluded based on clinical screening, to rule out the possibility that impaired perception of bladder signals was due to an organic issue caused by adhesions on the targeted organ (i.e. the bladder itself). Nevertheless, the exclusion of these patients limits the generalisability of our findings. Moreover, the use of hormonal contraception was assessed for the patients’ group but not for the control group. This could constitute a limitation by causing the two tested samples to be less homogeneous. However, further analyses (see Supplementary Materials) revealed no significant differences in interoceptive accuracy between patients with different hormonal treatments (i.e., progestin, oestrogen, or no medication), suggesting that hormonal contraception did not substantially affect our main findings.*

*In addition, it is important to consider the potential influence of co-occurring mental health disorders, on the observed differences in interoceptive accuracy between groups. Chronic health conditions like endometriosis are often associated with higher rates of anxiety and depression [73-75], which may also affect interoceptive processing [33]. Although our exclusion criteria included the presence of neurological or psychiatric conditions, we did not assess the possible presence of moderate mental health symptoms. Thus, we cannot rule out the possibility that patients with endometriosis suffered from subclinical depressive or anxious symptoms. Future studies should include questionnaires tackling mental health markers and, where possible, match groups on this variable to better disentangle the specific contributions of these factors to interoceptive accuracy.”*

*We hope that adding this paragraph we complied with the reviewer’s request.*

2. I would like to see some discussion of the issues with the waterload task, alongside the issues with the HCT.

*We thank the reviewer for requesting further details on the limitations of the Waterload task. Following the reviewer’s previous comments, we already included a paragraph concerning the general issues of the WTL in the discussion. Therefore, we assumed that the reviewer would like us to add further details concerning how these limitations could affect our results specifically. Thus, we edited the discussion paragraph on line 410, page 17, as follows: “[…] Moreover, both tasks have limitations that need to be acknowledged. The principal drawback of the gastric water load task is that it relies on self-reported fullness, which is subjective and might be influenced by cognitive biases, mood, and prior experiences [35,7**2].* *Thus, patients with endometriosis might report different sensations due to pain-related hypervigilance rather than their actual interoceptive accuracy. This could lead to overestimating or underestimating gastric sensations, making it difficult to tease apart true interoceptive accuracy from pain-related cognitive interference. Furthermore, chronic pelvic pain conditions like endometriosis are often associated with visceral hypersensitivity, which might amplify sensations of gastric discomfort. Patients could then experience gastric fullness at lower water volumes, not because of impaired interoception but due to visceral hypersensitivity. Moreover, since the test assesses fullness using water rather than solid food, its ecological validity is limited, making it less reflective of real-world eating behaviours. Lastly, individuals may interpret ‘maximum fullness’ differently, leading to variability in results [35]. […]”*

We hope that with this integration, we addressed the reviewer’s concerns.

3. I’m afraid the lack of a control task is unjustifiable – without these, you cannot know that differences between groups are due to interoceptive abilities. The fact that other studies in patients are also of poor quality is not a defence. The limitation should be revised to point out that no clear conclusions regarding interoceptive ability can be made without the use of control tasks as differences may be due to non-interoceptive factors. This point should be made clearly and explicitly, at the moment this is framed as a future direction rather than a critical limitation.

*We thank the reviewer again for pointing out this limitation. We rephrased the paragraph to make it clear that the lack of a control task constitutes a critical limitation. The paragraph at line 427, page 17, now reads as follows: “Another limitation of this study is the absence of control tasks to assess whether participants manifest non-interoceptive perceptual impairments. This is particularly relevant for individuals suffering from chronic pain, as they may exhibit broader perceptual or attentional deficits that could influence their interoception task performances. ~~Hence, future studies should include control tasks that help isolate interoceptive-specific impairments from more generalised perceptual~~* *~~deficits~~. Hence, considering this limitation, it may not be fully possible to draw firm conclusions regarding patients’ interoceptive impairments, as differences may be due to non-interoceptive factors.” Nevertheless, despite this limitation, our findings provide an important step forward in understanding interoception in this population, highlighting the need for future studies to incorporate control tasks to disentangle interoceptive and non-interoceptive contributions.*

4. Analysis of the urinary task. In the example provided, both participants show perfect consistency (their minimum stimulus is the same both times, suggesting that when asked to perceive the same amount, they are consistent (i.e. highly interoceptive). The discrimination between the minimum and maximum does not provide this as it would be influenced by bladder capacity etc., I appreciate the authors adding this in the supplement (where no differences were found), but I think this should really be included in the main text, in relation to relationships between tasks as well, and in the discussion the differences between the metrics should be clearly articulated with some discussion of what this means for interpretations.

*We thank the reviewer for highlighting this perspective on the analysis of the urinary task. We want to clarify that we conceptualised the Urinary Interoceptive Index as the result of the ratio between the mean of the two minimum stimuli and the maximum stimulus for different reasons. First, it reflects the idea of the gastric Water load by Van Dyck et al. (2016), where there are two different stimuli to reach and compare (i.e., satiety and fullness for the gastric water load and minimum and maximum stimuli for the urinary water load). Second, we do not believe that the index would be strongly influenced by bladder capacity: for the way we conceptualised the index, a participant with a small bladder will reach both the maximum and the minimum stimuli at lower millilitres of urine if compared to a participant with a bigger bladder, but the ratio will stay the same. Thus, participants are accurate in detecting the difference between the two urinary stimuli, their urinary index will result higher than participants who are not as accurate, regardless of the size of their bladder. Last, we think that comparing the minimum vs. maximum urinary stimuli is more ecological than just comparing the two minimum stimuli, as people in everyday life indeed perceive the urinary stimuli at different intensities, reflecting different urgencies.*

*Nevertheless, we think that, as suggested by the reviewer, adding the measure reflecting participants’ consistency in detecting their minimum stimuli to the results and the discussion would improve the manuscript from its current state.*

*As suggested by the reviewer, we moved the analysis concerning the comparison between the two minimum stimuli from the supplementary materials to the main text. Data Analysis and Results sections now read as follows:*

*Data analysis, “Relationship between the Interoceptive Tasks” paragraph, line 216, page 10:*

*“We also wanted to explore if participants’ consistency in discriminating the two minimum urinary stimuli correlated with their ability to detect gastric and cardiac stimuli, measured through the gastric Waterload test and the Heartbeat Counting Task, respectively. Thus, we ran two correlation analyses using a correlation package (v0.6.0; [52]) to test the relation between participants’ consistency, the Heartbeat Counting task, and the Two-step Water Load Test. ”*

*Data analysis, “Interoceptive accuracy and beliefs in patients” paragraph, line 234, page 11: “To test participants’ consistency in detecting the two minimum stimuli (i.e., void2 and void3) in the two groups, we performed two non-parametric t-tests.”*

*Results, “Relationship between the Interoceptive Tasks” paragraph, line 249, page 11:*

*“**The correlation analyses performed considering the whole sample between participants’ consistency in detecting their minimum urinary stimuli (measured as the difference between void2 and void3) and cardiac and gastric interoceptive tasks showed that participants’ consistency in detecting their minimum urinary stimuli did not correlate with participants’ ability to detect either their gastric (R=-0.096, p=0.46) or cardiac (R=-0.03, p=0.82) signals.”*

*Results, “Interoceptive accuracy and beliefs in patients” paragraph, line 294, page 13: “**We also performed two non-parametric t-tests to test whether void2 and void3 were consistently different in the two groups. Results showed that void2 did not significantly differ from void3 neither in the control group (W=300, p=0.171) nor in the patients' group (W=265, p=0.510).”*

*Consequently, we edited the Discussion to integrate the findings regarding participants’ consistency accordingly. The related discussion paragraphs at line 479, page 19, now read as follows:*

*“**In addition to the urinary interoceptive index, this study considered a measure of consistency in perceiving the minimum interoceptive stimulus (i.e., void2 and void3). When exploring participants’ consistency, the results show that both groups did not significantly differ in the amount of urine expelled during the minimum stimuli. In contrast, control participants were more accurate in perceiving the two different urinary stimuli (i.e., void1 vs. void2 and void3) than endometriosis patients. This suggests that endometriosis pain hypervigilance may selectively affect the ability to differentiate between interoceptive stimuli of varying intensities due to an increased attentional focus on urgency or discomfort. On the contrary, the perception of lower-intensity stimuli across repeated instances remains unaffected, as these may not trigger the same heightened salience.*

*Moreover, urinary consistency was not correlated to performance in the cardiac and gastric interoceptive tasks, suggesting that this measure captures a distinct facet of interoception. Conversely, the urinary interoceptive index and the gastric interoceptive index are correlated. Thus, it is possible that the discrimination between two stimulus intensities, rather than consistency in detecting the same stimulus intensity, may rely on shared mechanisms across gastric and urinary domains. These findings highlight the need for future research to consider interoception as a multidimensional construct, where different components may be differentially affected in clinical conditions such as chronic pain.”*

*We hope that with this integration, we have now answered the reviewer’s concerns.*

Reviewer #2: I have read the rebuttal and revised manuscript and am satisfied with the authors' changes.
Key concerns, including updating interoceptive terminology to align with the multidimensional framework by Suksasilp & Garfinkel (2022), clarifying the power analysis details, and providing further methodological and analytical clarifications regarding the urinary interoception task, have been adequately addressed.
The authors have also improved transparency in their reporting of the water load test, ensured inclusive language, and discussed potential limitations where appropriate.
The manuscript is engaging, and the results provide meaningful insights into interoceptive accuracy in patients with endometriosis across different bodily axes, contributing to interoception and chronic pain research.

*We thank the reviewer, and we are happy that our revisions to the manuscript answered the reviewer’s concerns.*
